# 

**Published:** 1891-09-26

**Authors:** 


PRICE ONE PENNY.

No. 261, Vol. X.—September 26th, 1891.

[Registered at the General Post Office as a Newspaper for transmission in the United Kingdom and Abroad.]

WITH EXTRA NURSING SUPPLEMENT.

Per Annum, 6s. 6d.; post free.

Monthly Parts, 6d.; post free 8d.

Ditto per Annum, 8s., post free.

### THE HOSPITAL

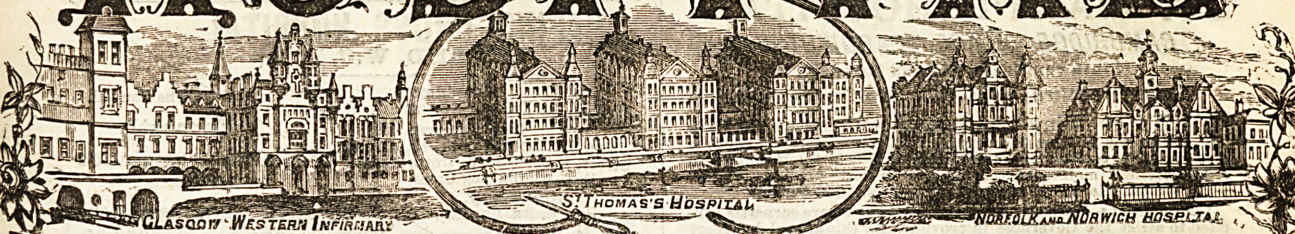

A WEEKLY JOURNAL OF

### Science, Medicine, Nursing and Philanthropy.

SATURDAY, SEPTEMBER 26th, 1891.

**Cycling and Health** . . . 299

**Passing Topics.**—Shall Nurses Cease to be Laywomen?—III. . . . 300

**The Doctor's Armchair.**—Village Anthropology . . . 301

**Some Ludicrous Aspects of Human Misery** . . . 302

**Everybody's Page** . . . 303

**Notes and News** . . . 304

**General Charities—Scraps and Gleanings** . . . 305

**Annotations.**—Honesty and the Professions—One's Own—The Last of the Demographers . . . 306

**The Practitioner's Mirror and Retrospect**—

MEDICINE.—Antiseptic Treatment of Typhoid Fever . . . 307

SURGERY.—Hæmaturia: The Diagnosis of its Cause, 308; Genu Valgum.—I. . . . 309

PRACTITIONERS' BOOKSHELF.—A Medical Bibliography . . . 309

NEW DRUGS, APPLIANCES, AND THINGS MEDICAL—New Nurses' Case . . . 309

**The Woman Question in India** . . . 310

**The Student of Medicine.**—Appointments—Vacancies—Pass Lists

EXTRA SUPPLEMENT—THE NURSING MIRROR.

**En Passant.**—Nightingale Nurses—Short Items—Nursing Uniforms—The Nurses' Directory—Nurses and Students—The Nurse and the Undertaker . . . cxlix

**Lectures on Surgical Ward Work and Nursing.**—By ALEX. MILES, M.D.Edin., F.R.C.S.E. Lecture XXXVI.—Saws . . . cl

**Where to Go** . . . cl

**Glasgow Royal Infirmary** . . . cl

**To Would-be Nurses—Notice** . . . cli

**For Reading to the Sick**—A Garland . . . cli

**Pleasures of the East** . . . clii

**Christmas Competitions** . . . clii

**A Bed for a Sick Nurse** . . . cliii

**Everybody's Opinion.**—Nurses and Rats—Drunken Nurses—Housekeeping . . . cliii

**Death in Our Ranks** . . . cliii

**Notes and Queries** . . . cliii

**Wants and Workers** . . . cliii

**Off Duty.**—Content to Follow (*concluded*) . . . cliv

**Amusements and Relaxation** . . . cliv

